# The long non-coding RNA ANRIL promotes proliferation and cell cycle progression and inhibits apoptosis and senescence in epithelial ovarian cancer

**DOI:** 10.18632/oncotarget.8744

**Published:** 2016-04-15

**Authors:** Jun-jun Qiu, Yan Wang, Ying-lei Liu, Ying Zhang, Jing-xin Ding, Ke-qin Hua

**Affiliations:** ^1^ Department of Gynecology, Obstetrics and Gynecology Hospital, Fudan University, Shanghai, China; ^2^ Department of Obstetrics and Gynecology of Shanghai Medical College, Fudan University, Shanghai, China; ^3^ Shanghai Key Laboratory of Female Reproductive Endocrine-Related Diseases, Fudan University, Shanghai, China; ^4^ Cancer Institute, Shanghai Medical College, Fudan University, Shanghai, China; ^5^ Department of Oncology, Shanghai Medical College, Fudan University, Shanghai, China; ^6^ Department of Gynecology, The Second Affiliated Hospital of Nantong University, Nantong, China

**Keywords:** ANRIL, proliferation, cell cycle, apoptosis, senescence

## Abstract

Antisense non-coding RNA in the INK4 locus (ANRIL) has been implicated in a variety of cancers. In the present study, we evaluated ANRIL expression in epithelial ovarian cancer (EOC) and defined its clinical implications and biological functions. ANRIL was overexpressed in EOC tissues relative to normal controls. Overexpression correlated with advanced International Federation of Gynecologists and Obstetricians stage and high histological grade. Multivariate analysis indicated that ANRIL is an independent prognostic factor for overall survival in EOC. Gain- and loss-of-function experiments demonstrated that ANRIL promotes EOC cell proliferation both *in vitro* and *in vivo*. The proliferative effect was linked to the promotion of cell cycle progression and inhibition of apoptosis and senescence. Down-regulation of P15^INK4B^ and up-regulation of Bcl-2 by ANRIL may partially explain ANRIL-induced EOC cell proliferation. This study is the first to establish that ANRIL promotes EOC progression and is a potential prognostic biomarker.

## INTRODUCTION

Epithelial ovarian cancer (EOC) is one of the most aggressive malignancies [1–3]. The prognosis of EOC is very poor, primarily because it is typically diagnosed at an advanced stage and because of the lack of efficacious therapies [3]. Despite extensive clinical and basic research, the overall survival (OS) rate has not improved [4, 5]. A better understanding of the molecular mechanisms involved in EOC progression may facilitate the identification of novel markers and effective therapeutic strategies that can prolong survival and improve patient outcomes.

Mounting evidence indicates that the molecular mechanisms of carcinogenesis and cancer progression involve not only to protein-coding genes, but also non-coding RNAs. Long non-coding RNAs (lncRNAs, > 200 nucleotides) were initially thought to be the “dark matter” of the genome but have recently emerged as critical components of the cancer transcriptome [6, 7]. Many lncRNAs control carcinogenesis and cancer progression, and potentially represent a new avenue for cancer research [8–17]. Elucidation of the roles of these lncRNAs will provide insight into the molecular biology underlying cancer initiation and progression.

Recently, antisense non-coding RNA in the INK4 locus (ANRIL) has garnered substantial attention. ANRIL is transcribed as a 3.8-kb lncRNA in the antisense orientation of the INK4b/ARF/INK4a gene cluster [18]. ANRIL was initially identified in a genetic analysis of familial melanoma patients with neural tumors [19]. It was subsequently determined to be independently associated on a genome-wide level with several other forms of cancer including breast cancer [20], pancreatic carcinoma [21], nasopharyngeal carcinoma [22], basal cell carcinoma [23], glioma [24], leukemia [25], prostate cancer [26], esophageal squamous cell carcinoma [27] and gastric cancer [28]. We recently linked ANRIL to metastasis in serous ovarian cancer (SOC), a histotype of EOC [29]. However, the role of ANRIL in EOC has not been determined and an association between ANRIL and other aspects of EOC progression in addition to metastasis has not been demonstrated.

In the present study, we investigated the expression pattern, clinical significance, and biological functions of ANRIL in EOC including proliferation, cell cycle progression, apoptosis, and senescence. We demonstrated that ANRIL levels were elevated in EOC tissues compared with normal controls and that ANRIL serves as an independent predictor of overall survival (OS) in EOC patients. We also determined that ANRIL promotes EOC cell proliferation both *in vitro* and *in vivo*. This effect on proliferation was associated with the promotion of cell cycle progression and inhibition of both apoptosis and senescence. Down-regulation of P15^INK4B^ and up-regulation of Bcl-2 by ANRIL may partially explain ANRIL-induced EOC cell proliferation. Our study is the first to establish that ANRIL contributes to EOC progression and that ANRIL has the potential to be a novel biomarker for predicting poor survival in EOC patients.

## RESULTS

### Overexpression of ANRIL is correlated with FIGO stage, histological grade, and poor prognosis in EOC

**Figure 1:**
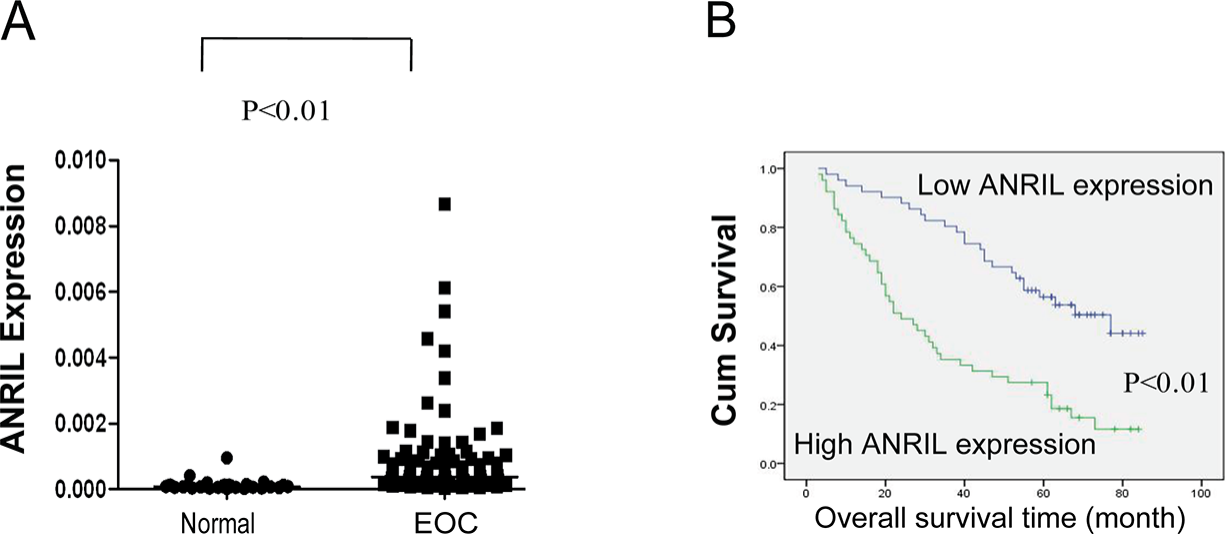
Relative ANRIL expression levels and their association with poor prognosis in EOC (**A**) Relative ANRIL expression levels in EOC and normal ovarian tissues. (**B**) Kaplan-Meier analysis of OS was performed based on ANRIL expression levels.

We showed that ANRIL expression was significantly elevated in 102 EOC tissues compared with 30 noncancerous tissues using qRT-PCR (*P* < 0.01; Figure 1A). The median relative ANRIL expression value was used as a cut-off [14] to divide the 102 EOC patients into a high-ANRIL group (*n* = 51; an ANRIL level ≥ the median value) and a low-ANRIL group (*n* = 51; an ANRIL level < the median value). An examination of the correlation between ANRIL expression and clinicopathological features revealed that increased ANRIL expression was correlated with advanced FIGO stage and high histological grade, but not with age, histological type, residual tumor diameter, CA-125 level, or ascites (Table 1). These results suggested that ANRIL overexpression was associated with a more malignant ovarian cancer phenotype.

**Table 1:** Association of ANRIL expression with clinicopathological variables in EOC patients

Variables	Low ANRIL expression (*n* = 51)	High ANRIL expression(*n* = 51)	*P*
	*n* (%)	*n* (%)	
Age (years)			
< 50	18 (43.9)	23 (56.1)	0.313
≥ 50	33 (54.1)	28 (45.9)	
Histological subtype			
Serous	31 (45.6)	37 (54.4)	0.208
Other	20 (58.8)	14 (41.2)	
FIGO stage			
I–II	25 (75.8)	8 (24.2)	< 0.001
III–IV	26 (37.7)	43 (62.3)	
Histological grade			
G1-G2	25 (67.6)	12 (32.4)	0.007
G3	26 (40.0)	39 (60.0)	
Residual tumor diameter (cm)			
< 1	24 (47.2)	18 (52.8)	0.385
≥ 1	27 (56.7)	33 (43.3)	
CA125 level (U/ml)			
< 600	38 (57.1)	24 (42.9)	0.227
≥ 600	13 (45.0)	27 (55.0)	
Ascites			
< 100	24 (58.5)	17 (41.5)	0.157
≥ 100	27 (44.3)	34 (55.7)	

To evaluate survival, univariate log-rank tests and multivariate Cox regression analyses were performed. As shown in Figure 1B and Table 2, OS was significantly shorter for patients with high ANRIL expression compared to those with low expression (*P* < 0.01). Additionally, the multivariate analyses revealed that ANRIL expression, FIGO stage, and histological grade were independent predictors of OS (*P* < 0.01, Table 2). Based on these data, we concluded that ANRIL could serve as a predictive biomarker for EOC outcome and that ANRIL overexpression may contribute to EOC progression.

**Table 2:** Univariate and multivariate analysis of overall survival in 102 EOC patients

Variables	Univariate analysis		Multivariate analysis					
	Overall survival (months)	*P*	Overall survival					
	Mean ± SE		β	SE	Wald	*P*	Exp (β)	95% CI
Age (years)								
< 50	45.60 ± 4.37	0.634	—	—	—	—	—	—
≥ 50	49.01 ± 3.81		—	—	—	—	—	—
Histological subtype								
Serous	48.56 ± 3.45	0.839	—	—	—	—	—	—
Other	47.36 ± 5.51		—	—	—	—	—	—
CA125 level (U/ml)								
< 600	53.86 ± 4.44	0.131	—	—	—	—	—	—
≥ 600	43.97 ± 3.81		—	—	—	—	—	—
Ascites								
< 100	55.23 ± 4.49	0.075	—	—	—	—	—	—
≥ 100	43.28 ± 3.75		—	—	—	—	—	—
FIGO stage								
I–II	77.51 ± 2.48	< 0.001	—	—	—	—	—	—
III–IV	33.68 ± 2.84		1.958	0.461	18.084	< 0.001	7.089	2.874–17.482
Histological grade								
G1–G2	66.45 ± 4.34	< 0.001	—	—	—	—	—	—
G3	37.43 ± 3.19		0.865	0.319	7.348	0.007	2.375	1.271–4.439
Residual tumor								
diameter (cm)								
< 1	52.39 ± 3.60	0.01	—	—	—	—	—	—
≥ 1	38.44 ± 4.65		0.478	0.270	3.129	0.077	1.613	0. 950–2.738
ANRIL expression								
Low	61.71 ± 3.68	<	—	—	—	—	—	—
High	34.62 ± 3.73	0.001	0.656	0.278	5.574	0.018	1.928	1.118–3.324

β: Regression coefficient; SE: Standard error; CI: confidence interval.

### ANRIL knockdown inhibits EOC cell proliferation *in vitro*

**Figure 2:**
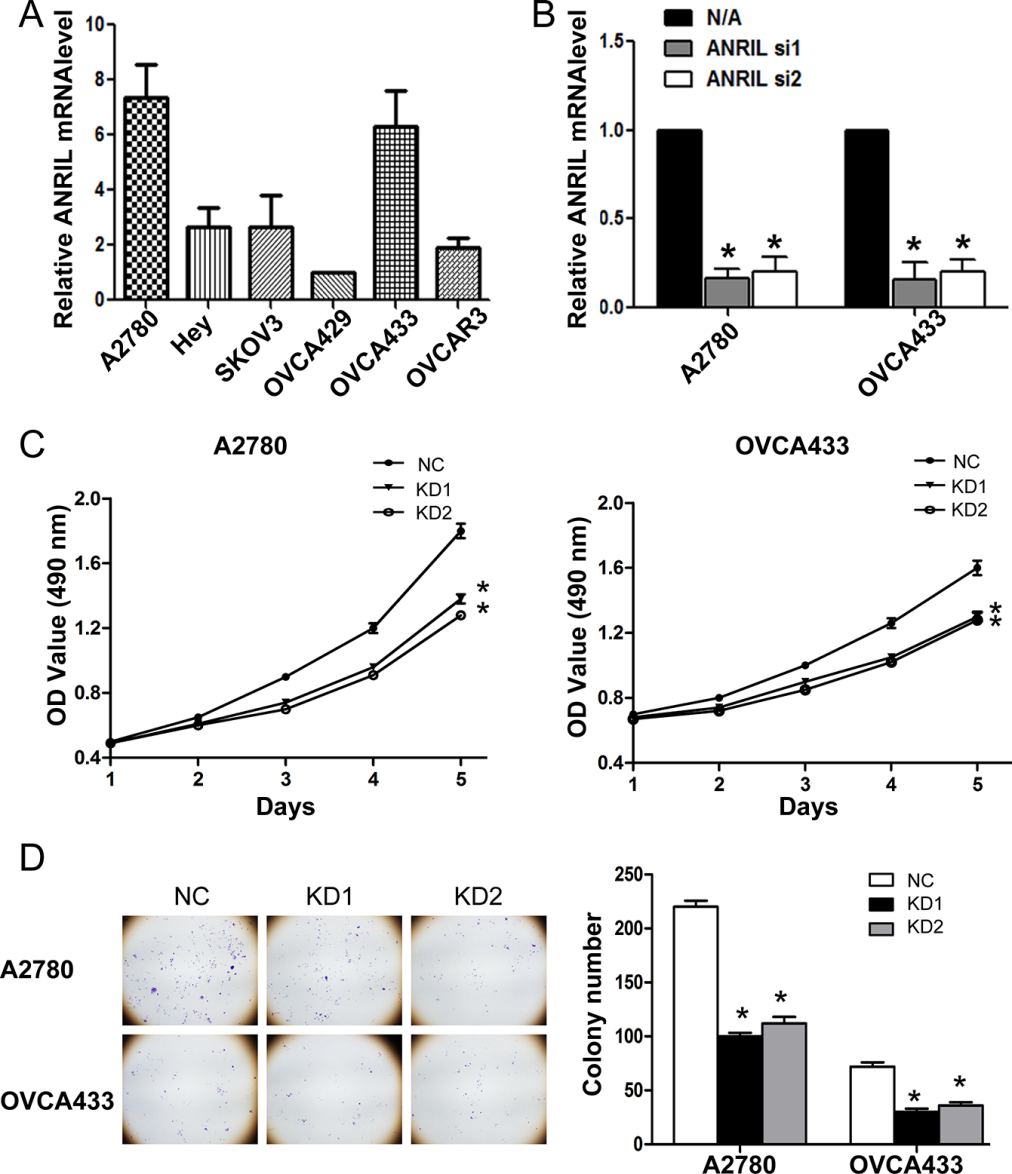
ANRIL knockdown inhibits the proliferation of A2780 and OVCA433 cells (**A**) Relative ANRIL expression levels in EOC cell lines (A2780, Hey, SKOV3, OVCA429, OVCA433, and OVCAR3). (**B**) Relative ANRIL expression levels in A2780 and OVCA433 cells transfected with si-NC or ANRIL siRNAs. *P < 0.05. (**C**) MTT assays were performed to evaluate the proliferation of A2780-KD and OVCA433-KD cells compared to controls. The data represent the mean ± standard deviation (SD) of three independent experiments. Bars denote the SD. *P < 0.05. (**D**) Representative colony formation assay results for A2780-KD and OVCA433-KD cells and corresponding controls. *P < 0.05.

Based on the association of ANRIL with a more malignant cancer phenotype in EOC, we hypothesized that ANRIL was involved in EOC cell proliferation. We therefore examined ANRIL levels in six EOC cell lines. Of these cell lines, the A2780 and OVCA433 cells exhibited the highest ANRIL expression (Figure 2A) and were selected for further loss-of-function experiments. Because both siRNAs efficiently silenced ANRIL expression in these cell lines (Figure 2B), we designed lentiviral vectors to establish stable ANRIL knockdown cell lines (A2780-KD1, A2780-KD2, OVCA433-KD1, and OVCA433-KD2 cells) and the corresponding controls (A2780-NC and OVCA433-NC cells).

To assess the effects of ANRIL on EOC cell proliferation *in vitro*, we performed MTT and colony formation assays. MTT assays demonstrated that A2780-KD and OVCA433-KD cells proliferated more slowly than A2780-NC and OVCA433-NC cells (Figure 2C). In addition, there were fewer A2780-KD and OVCA433-KD colonies compared to A2780-NC and OVCA433-NC colonies (Figure 2D). These results suggested that ANRIL knockdown inhibited EOC cell proliferation *in vitro*.

### ANRIL knockdown inhibits cell cycle progression and promotes apoptosis and senescence

**Figure 3:**
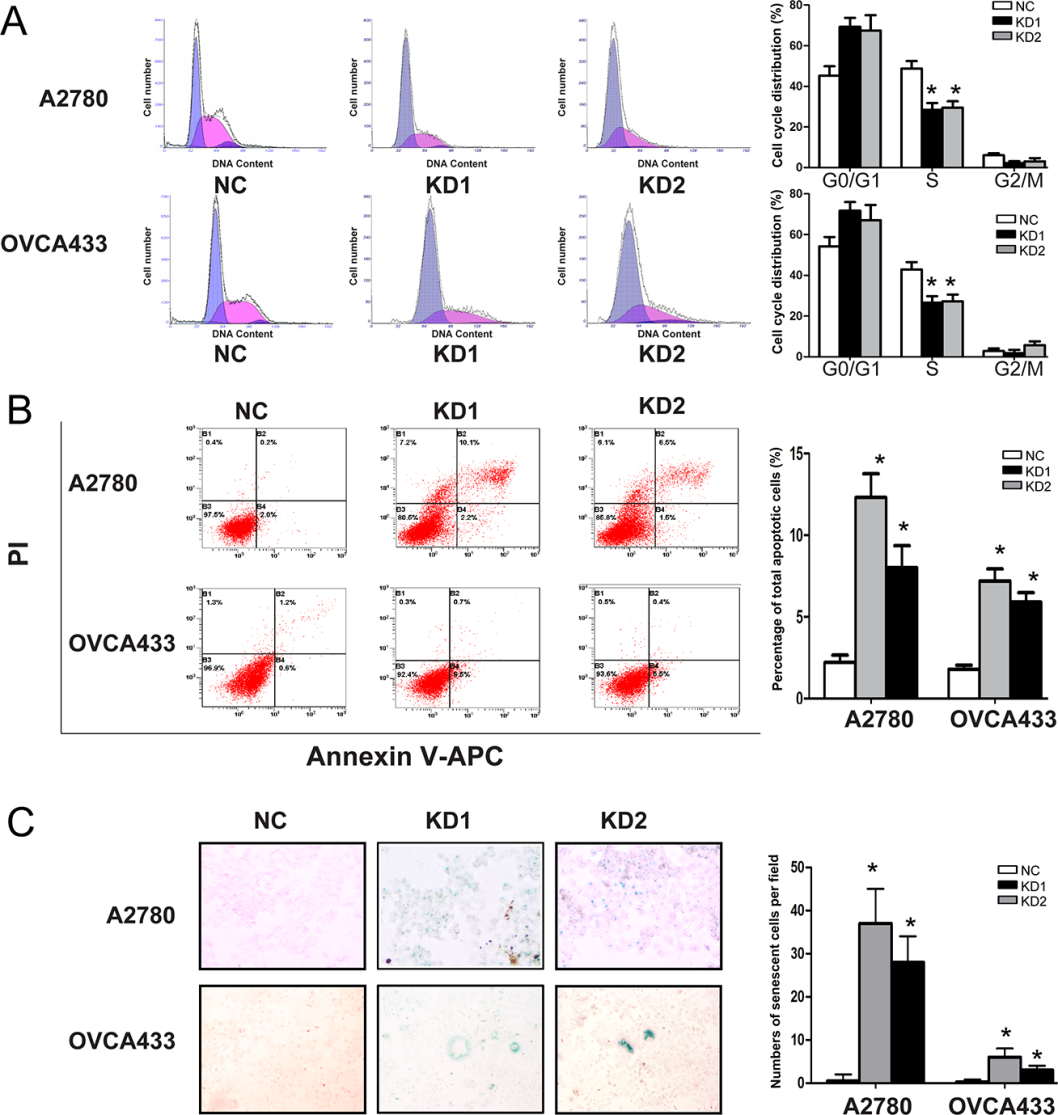
ANRIL knockdown inhibits cell cycle progression and promotes apoptosis and senescence in A2780 and OVCA433 cells (**A**) Cell cycle analysis was performed using flow cytometry. The bar graph on the right presents the percentage of cells in the G0-G1, S, or G2-M phases of the cell cycle. Representative histograms are presented on the left. The results shown are representative of three independent experiments. *P < 0.05. (**B**) Apoptosis was assessed using flow cytometry. The bar graph on the right presents the percentage of apoptotic cells. Representative quadrant figures are presented on the left. The results shown are representative of three independent experiments. *P < 0.05. (**C**) Cells were stained with the senescence marker β-galactosidase. The blue staining around the nucleus in A2780-KD and OVCA433-KD cells indicates cellular senescence. *P < 0.05.

To explore the cause of the growth inhibition following ANRIL-knockdown, we evaluated the effect of ANRIL on cell cycle progression. Flow cytometry results indicated that the proportion of cells in S phase decreased in both A2780-KD and OVCA433-KD cells compared with the corresponding controls (Figure 3A), suggesting that ANRIL knockdown blocked cell cycle progression.

Given that a decrease in cell death (including cell death due to apoptosis) is one of the primary mechanisms underlying tumor growth, we evaluated the effect of ANRIL on apoptosis. ANRIL knockdown increased the total number of apoptotic cells in both the A2780-KD and OVCA433-KD cell lines compared to the corresponding controls (Figure 3B), indicating that ANRIL knockdown could promote apoptosis. We also investigated the effect of ANRIL on cell senescence since inducing senescence is an important strategy for arresting proliferation of oncogenic cells. The proportion of cells that were positive for β-galactosidase activity, an indicator of cell senescence, was increased in the A2780-KD and OVCA433-KD cell lines compared to the corresponding controls (Figure 3C), suggesting that ANRIL silencing may promote senescence. These results demonstrated that the inhibitory effect on proliferation induced by ANRIL silencing was partially due to the inhibition of cell cycle progression and the promotion of apoptosis and senescence.

### Overexpression of ANRIL enhances EOC cell proliferation and cell cycle progression and inhibits apoptosis and senescence

**Figure 4:**
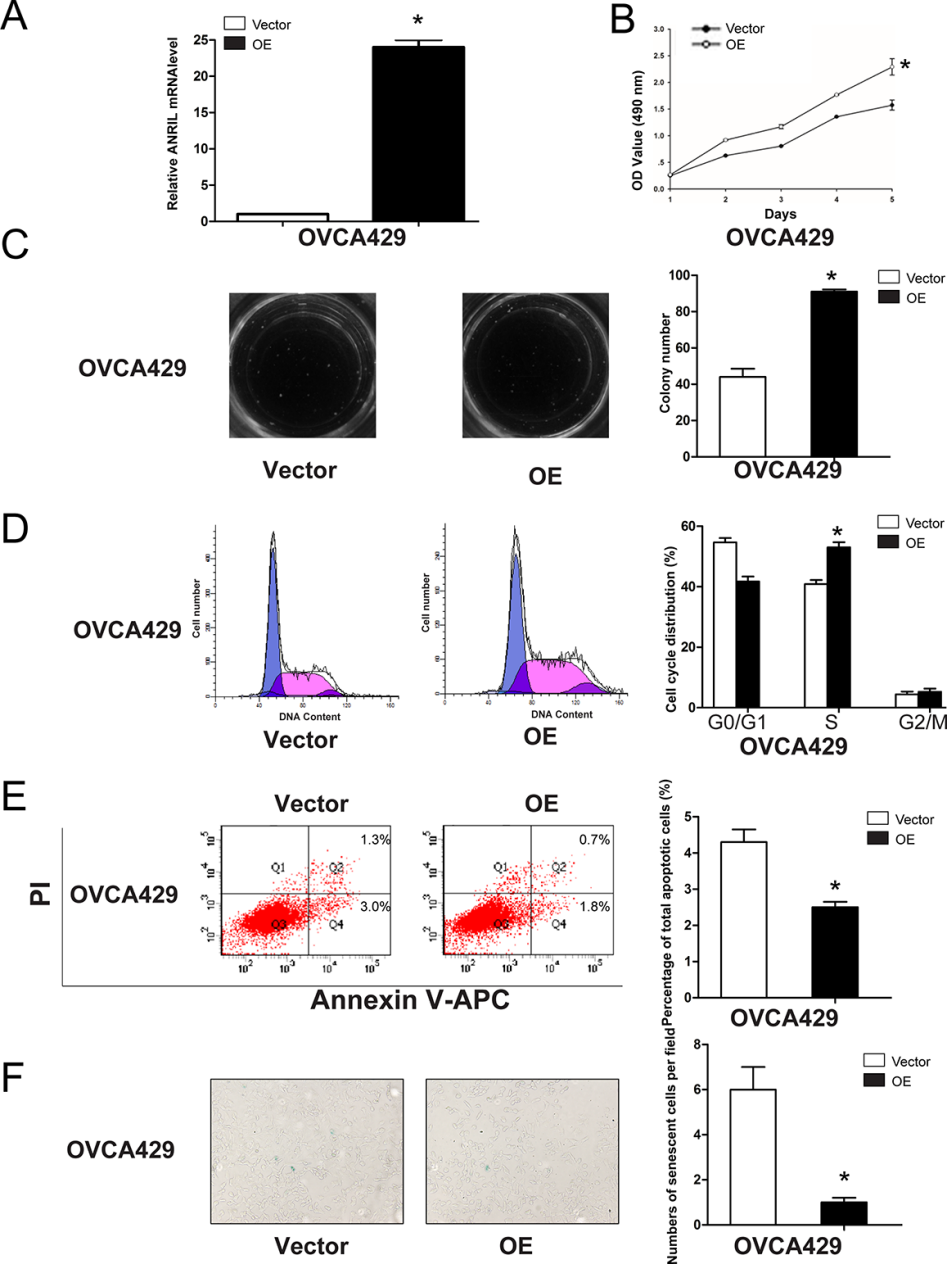
Overexpression of ANRIL promotes EOC cell proliferation and cell cycle progression, and inhibits apoptosis and senescence in OVCA429 cells (**A**) Relative ANRIL expression levels in OVCA429-OE cells and control cells. *P < 0.05. (**B**) MTT assays were performed to evaluate the proliferation of OVCA429-OE and OVCA429-Vector cells. *P < 0.05. (**C**) Representative colony formation assay results for OVCA429-OE and OVCA429-Vector cells. *P < 0.05. (**D**) Cell cycle analysis was performed using flow cytometry in OVCA429-OE and OVCA429-Vector cells. The bar graph on the right presents the percentage of cells in the G0–G1, S, or G2-M phases of the cell cycle. *P < 0.05. (**E**) Apoptosis was assessed using flow cytometry. The bar graph on the right presents the percentage of apoptotic cells. *P < 0.05. (**F**) OVCA429-OE and OVCA429-Vector cells were stained with β-galactosidase. The blue staining around the nucleus indicates cellular senescence. *P < 0.05.

We next performed gain-of-function experiments to further elucidate the effects of ANRIL on proliferation, cell cycle progression, apoptosis, and senescence. We established stable OVCA429-OE cells because this cell line exhibited the lowest ANRIL expression of the cell lines examined (Figure 2A). The efficiency of ANRIL overexpression was confirmed using qRT-PCR (Figure 4A). MTT and colony formation assays showed that overexpression of ANRIL increased OVCA429 cell proliferation (Figure 4B, 4C). Flow cytometry analysis revealed that overexpression of ANRIL promoted cell cycle progression by enhancing the proportion of OVCA429 cells in S phase (Figure 4D). Apoptosis assays showed that overexpression of ANRIL decreased the total number of apoptotic OVCA429 cells (Figure 4E). Senescence assays demonstrated that population of cells positive for β-galactosidase activity decreased in OVCA429-OE cells (Figure 4F). These results indicated that overexpression of ANRIL enhanced EOC cell proliferation partially through the promotion of cell cycle progression and the inhibition of apoptosis and senescence.

### P15 and Bcl-2 are key genes downstream of ANRIL that promote EOC cell proliferation

**Figure 5:**
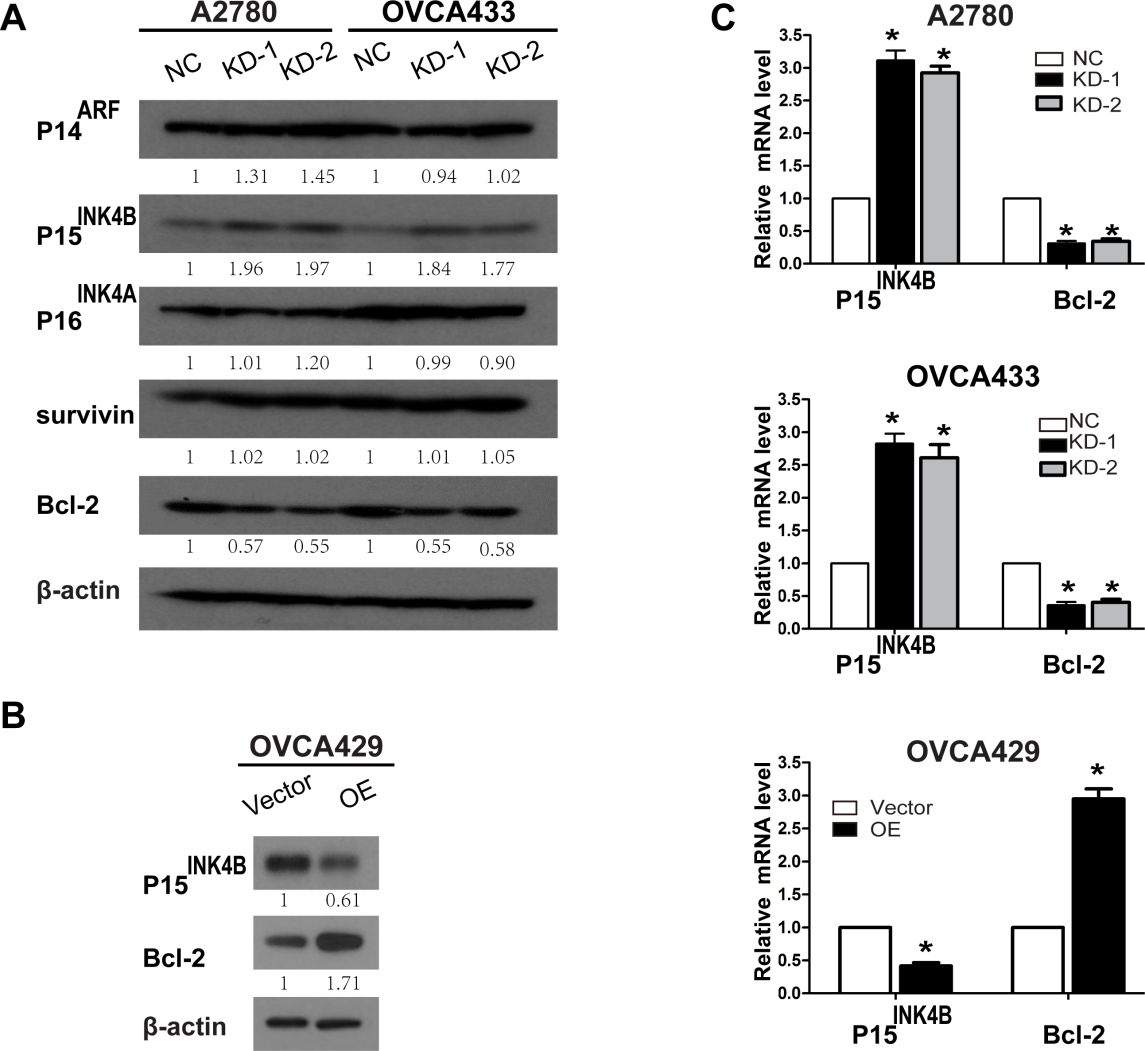
Knockdown and overexpression of ANRIL alters P15^INK4B^ and Bcl-2 expression (**A**) Western blots showing that ANRIL knockdown increases P15^INK4B^ and decreases Bcl-2 levels in A2780 and OVCA433 cells. (**B**) Western blots showing that overexpression of ANRIL decreases P15^INK4B^ and increases Bcl-2 protein levels in OVCA429 cells. (**C**) Increased P15^INK4B^ and decreased Bcl-2 mRNA levels were detected by qRT-PCR in A2780-KD and OVCA433-KD cells while decreased P15^INK4B^ and increased Bcl-2 mRNA levels were detected in OVCA429-OE cells.

ANRIL inhibits neighboring tumor suppressors (P14^ARF^, P15^INK4B^, and P16^INK4A^), which can impair cell cycle progression and influence key physiological processes including senescence and apoptosis [18, 30]. Thus, we examined whether ANRIL altered the expression of P14^ARF^, P15^INK4B^, and P16^INK4A^ in EOC cells. Western blotting demonstrated that ANRIL knockdown in A2780 and OVCA433 cells increased P15^INK4B^ but not P16^INK4A^ or P14^ARF^ protein levels (Figure 5A). We also examined the expression of Bcl-2 and survivin, since both are central regulators of apoptosis and contribute to tumor progression [31, 32]. Western blotting demonstrated that ANRIL silencing in A2780 and OVCA433 cells decreased the level of Bcl-2 but not survivin (Figure 5A). We then analyzed the expression of P15^INK4B^ and Bcl-2 in OVCA429-OE cells by western blotting. ANRIL overexpression decreased P15^INK4B^ but increased Bcl-2 protein levels (Figure 5B). Consistent with these results, increased P15^INK4B^ and decreased Bcl-2 mRNA levels were detected in A2780-KD and OVCA433-KD cells, while decreased P15^INK4B^ and increased Bcl-2 mRNA levels were detected in OVCA429-OE cells (Figure 5C). These results suggested that ANRIL promoted EOC cell proliferation in part by decreasing P15^INK4B^ and increasing Bcl-2 levels.

Interestingly, we found that ANRIL knockdown in A2780 and OVCA433 cells increased the levels of apoptosis markers such as caspase-9 and caspase-3, but not caspase-7. In contrast, ANRIL overexpression decreased the levels of caspase-9 and caspase-3 but not caspase-7 (Supplementary Figure S1). These results are consistent with previous studies, which reported that ANRIL inhibits caspase-9 and caspase-3 expression in bladder cancer [33].

### ANRIL contributes to EOC tumor growth *in vivo*

**Figure 6:**
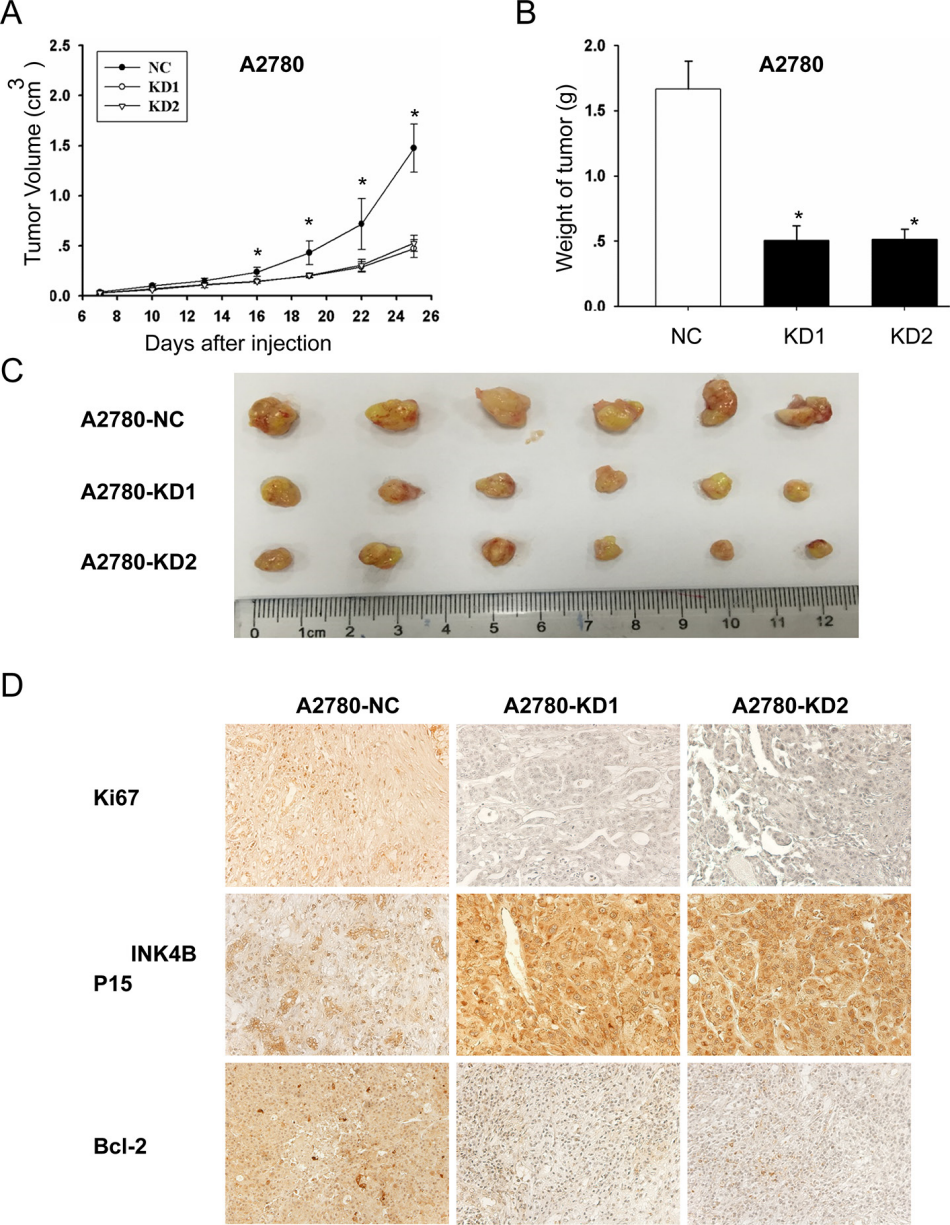
ANRIL knockdown inhibits A2780 cell proliferation *in vivo* (**A**) Growth curves of tumor xenografts. The volumes of tumors originating from A2780-KD1 and A2780-KD2 cells were substantially smaller than those originating from A2780-NC cells, and the size difference between the two groups increased over time (six mice per group; *P < 0.05). (**B**) The weights of the A2780-NC tumors were significantly greater than those of the A2780-KD1 and A2780-KD2 tumors. *P < 0.05. (**C**) Representative images of tumors in nude mice after subcutaneous injection of A2780-NC, A2780-KD1, and A2780-KD2 cells. (**D**) Immunohistochemical staining showing that tumors originating from A2780-KD1 and A2780-KD2 cells had increased P15^INK4B^ and decreased ki67 and Bcl-2 levels compared to those originating from A2780-NC cells.

We further examined the effects of ANRIL on EOC cell proliferation *in vivo*. A2780-KD1, A2780-KD2, and A2780-NC cells were injected into nude mice and tumor growth was analyzed. The tumors formed by A2780-KD1 and A2780-KD2 cells were smaller in both size and weight compared to A2780-NC tumors (Figure 6A, 6B and 6C). Immunohistochemical staining revealed that tumor nodules originating from A2780-KD1 and A2780-KD2 cells had decreased ki67, increased P15^INK4B^, and decreased Bcl-2 expression compared to nodules originating from A2780-NC cells (Figure 6D). These results were consistent with the *in vitro* studies and confirmed that ANRIL contributed to EOC tumor growth *in vivo* in part through down-regulation of P15^INK4B^ and up-regulation of Bcl-2.

## DISCUSSION

The involvement of lncRNAs in tumor processes such as proliferation, apoptosis, and metastasis reinforces the roles of these transcripts in cancer progression and suggests that they may be prognostic biomarkers. Indeed, HOTAIR was identified as a prognostic marker of metastasis in diverse human cancers [34–38]. MALAT-1 promotes metastasis and serves as a prognostic indicator in lung cancer [10]. GAS5 inhibits proliferation and serves as a prognostic indicator in hepatocellular and colorectal cancer [39, 40]. Here, we have reported the first evidence indicating that ANRIL is overexpressed in EOC tissues. ANRIL overexpression was correlated with advanced FIGO stage and high histological grade. Moreover, univariate and multivariate survival analyses demonstrated that ANRIL could serve as an independent prognostic indicator. These clinical findings highlight the potential value of ANRIL as a novel prognostic marker for EOC and suggest that ANRIL contributes to EOC progression.

ANRIL has been implicated in many human solid carcinomas [19–25, 27, 28]. Although our previous study linked ANRIL to metastasis in SOC, it was not clear whether ANRIL contributed to EOC and whether it was associated with other aspects of EOC progression in addition to metastasis. In the present study, we showed that ANRIL depletion in A2780 and OVCA433 cells inhibited proliferation, delayed cell cycle progression, and promoted apoptosis and senescence. In contrast, overexpression of ANRIL in OVCA429 cells enhanced proliferation, promoted cell cycle progression, and inhibited apoptosis and senescence. Additionally, *in vivo* experiments confirmed that ANRIL knockdown inhibited tumor growth in nude mice. These data suggest that ANRIL is an important factor in promoting EOC growth and that ANRIL likely promotes cell cycle progression and inhibits apoptosis and senescence to drive tumor growth.

The downstream molecular events by which ANRIL promotes EOC cell proliferation are not yet clear. ANRIL inhibits P14^ARF^ (a regulator of the p53 pathway), P15^INK4B^, and P16^INK4A^ (two cyclin-dependent kinase inhibitors), which are neighboring tumor suppressors [18]. P15^INK4B^ has a well-described role in proliferation, cell cycle progression, and replicative senescence [18, 30]. Consistent with these previous findings, our data demonstrated that ANRIL decreased P15^INK4B^ protein and mRNA levels, suggesting that ANRIL may promote EOC cell cycle progression, inhibit senescence, and enhance proliferation partially through decreasing P15^INK4B^ levels. Given the evidence suggesting that ANRIL can also act on specific genes independently of P14^ARF^/P15^INK4B^/P16^INK4A^ [41, 42], we investigated whether ANRIL altered the expression of Bcl-2 and survivin, two central regulators of proliferation and apoptosis. As expected, ANRIL silencing decreased Bcl-2 protein and mRNA levels while overexpression of ANRIL increased Bcl-2 protein and mRNA levels. These results are consistent with previous data indicating that ANRIL knockdown repressed proliferation and promoted apoptosis in bladder cancer by reducing Bcl-2 levels [33]. *In vivo* experiments confirmed that ANRIL promoted EOC tumor growth in part by decreasing P15^INK4B^ and increasing Bcl-2 levels. Insight into the mechanisms by which ANRIL alters P15^INK4B^ and Bcl-2 expression was provided by a previous study that showed that ANRIL depletion could disrupt SUZ12, a component of the polycomb repressive complex 2 (PRC2), by binding to the P15^INK4B^ locus and increasing P15^INK4B^ expression [43]. Additionally, a recent study reported that P15^INK4B^ down-regulated Bcl-2 expression in chronic myeloid leukemia cells [44]. Collectively, our data and the previous findings suggest that P15^INK4B^ and Bcl-2 are key genes downstream of ANRIL that promote EOC cell proliferation. A limitation of the present study was that we did not investigate the exact mechanism involving “ANRIL-P15^INK4B^-Bcl-2”. Thus, further studies are required to elucidate the underlying molecular mechanisms.

In summary, our clinical data demonstrated that ANRIL was overexpressed in EOC, which was correlated with FIGO stage, and could serve as an independent predictor for OS. Moreover, gain- and loss-of-function studies demonstrated that ANRIL promoted EOC cell proliferation both *in vitro* and *in vivo*, and that the proliferative effect was linked to the promotion of cell cycle progression and inhibition of apoptosis and senescence. Finally, we demonstrated that ANRIL decreased P15^INK4B^ and increased Bcl-2 expression, which may partially account for ANRIL-induced EOC cell proliferation. Our study is the first to establish that ANRIL promotes EOC progression and that it may be a biomarker that can be used to predict poor survival.

## MATERIALS AND METHODS

### Patients and tissue samples

This study was authorized by the Research Ethics Committee of Fudan University, China. All patients agreed to the procedure and signed consent forms. All specimens were handled according to ethical and legal standards.

The study consisted of 102 EOC tissues, including 68 SOC tissues that were part of our previous study [29], 20 mucinous ovarian cancer tissues, and 14 endometrioid ovarian cancer tissues that were obtained from the Tissue Bank of the Obstetrics and Gynecology Hospital of Fudan University. All specimens were surgically resected from patients who were admitted to the Department of Gynecology at the Obstetrics and Gynecology Hospital of Fudan University between August 2005 and December 2008. All EOC tissues were selected from patients who (a) did not receive preoperative radiotherapy, chemotherapy, or hormonal therapy; and (b) did not have borderline ovarian tumors or two or more different malignancies. Thirty normal ovarian epithelial tissues were obtained from participants diagnosed with uterine fibroids who were scheduled to undergo a hysterectomy and oophorectomy. Tissues were selected from participants who did not have a history of ovarian cysts, ovarian pathology, or ovarian surgery. All samples were immediately frozen in liquid nitrogen and stored at −80°C until use.

The clinicopathological characteristics of all 102 EOC patients including age, histological subtype, International Federation of Gynecologists and Obstetricians (FIGO) stage, histological grade, residual tumor diameter, serum CA-125 level, and the presence of ascites were extracted from medical charts and original pathology reports. The tumor stage and histological grade diagnoses were determined according to the criteria established by the FIGO and the World Health Organization. Follow-up data were obtained by reviewing outpatient charts or via correspondence. OS was defined as the interval between the date of surgery and the date of death or the end of follow-up (January 2013).

### Cell lines and cell culture

The SKOV3, OVCAR3, A2780, Hey, OVCA429, and OVCA433 human EOC cell lines were gifts from the University of Texas M.D. Anderson Cancer Center (Houston, TX, USA). A2780, Hey, SKOV3, OVCA429, and OVCA433 cells were cultured in RPMI-1640 (Gibco, Gaithersburg, MD, USA). OVCAR3 cells were maintained in McCoy's 5A (modified) medium in humidified air at 37°C in a 5% CO_2_ atmosphere. All media were supplemented with 10% fetal bovine serum (FBS; Gibco), 2 mM L-glutamine, 100 U/mL penicillin, and 100 mg/mL streptomycin.

### Quantitative real-time polymerase chain reaction (qRT-PCR)

Total RNA was isolated from tissues or cells using TRIzol reagent (Invitrogen, Carlsbad, CA, USA) and reverse transcribed into cDNA using an ExScript RT-PCR kit (TaKaRa, Otsu, Japan) according to the manufacturer's instructions. The qRT-PCR reactions were performed using an ABI7500 System (Applied Biosystems, Foster City, CA, USA) and SYBR Green PCR Master Mix (TaKaRa). ANRIL expression was measured using the following primers: forward, 5′-TGTACTTAACCACTGGACTACCTGCC-3′ and reverse, 5′-CATTCTGATTCAACAGCAGAGATCAAAG-3′. GAPDH expression was used as an internal control and was measured with the following primers: forward, 5′-GTCAACGGATTTGGTCTGTATT-3′ and reverse, 5′-AGTCTTCTGGGTGGCAGTGAT-3′. All assays were performed in triplicate. Statistical analyses of the results were performed using the 2^−ΔΔCt^ relative quantification method.

### Establishment of stable cell lines in which ANRIL was overexpressed or knocked down

The two siRNA sequences were the following: 5′-GCAAGAAACATTGCTGCTAGC-3′ (ANRIL-si1) and 5′-GCCCAATTATGCTGTGGTAAC-3′ (ANRIL-si2). Lentiviral vectors encoding shRNA were designed based on the sequences of ANRIL-si1 and ANRIL-si2 to knock down ANRIL expression (ANRIL-knockdown, KD1 and KD2). These vectors were constructed by Hanyin Co. (Shanghai, China). The recombinant lentiviruses (KD1 and KD2) and the negative control (NC) lentivirus (Hanyin Co.) were prepared and titered to 10^9^ transfection units (TU)/mL. To obtain stable cell lines, A2780 and OVCA433 cells were seeded in six-well plates and infected with virus and polybrene the following day. Positive clones were selected with puromycin (2 μg/mL and 5 μg/mL for A2780 and OVCA433 cells, respectively) for 14 days to establish the following new stable cell lines: A2780-NC, A2780-KD1, A2780-KD2, OVCA433-NC, OVCA433-KD1, and OVCA433-KD2 cells. Additionally, the lentiviruses expressing the ANRIL sequence (ANRIL-overexpression, OE) and the negative control lentivirus (Vector) were constructed by Hanyin Co. (Shanghai, China). ANRIL-OE and control stable cell lines (OVCA429-OE, OVCA429-vector cells) were then established. The efficiency of ANRIL knockdown and overexpression was confirmed by qRT-PCR.

### Cell proliferation assays

A total of 3,000 A2780 cells, 1,000 OVCA433 cells, and 1,000 OVCA429 cells in 200 μL of medium were incubated in 96-well culture plates. Cell proliferation was assayed using a 3-(4, 5-dimethylthiazol-2-yl)-2, 5-diphenyltetrazolium bromide (MTT) kit (Sigma-Aldrich, St. Louis, MO, USA) and a Synergy H4 Hybrid Reader. Briefly, the culture medium was removed after 1, 2, 3, 4, and 5 days, and 0.5 mg/mL MTT in 200 μL of medium was added to each well and incubated for 4 h. The cells were then treated with 150 μL DMSO for 10 min, and the optical density (OD) measured at 540 nm. Each experiment was repeated in triplicate.

### Colony formation assays

A total of 500 A2780 cells, 150 OVCA433 cells, and 150 OVCA429 cells were plated in six-well plates. Duplicate cultures of each cell type were maintained at 37°C in a 5% CO_2_ atmosphere, and fresh medium was added every 3 days. After 2 weeks, colonies consisting of > 50 cells in each well were counted. Each experiment was repeated in triplicate.

### Cell cycle and apoptosis analysis

For cell cycle analysis, 2 × 10^6^ cells were harvested, fixed with 4 mL of cold 75% ethanol at −20°C overnight, and washed twice with PBS. The cells were then resuspended in 500 μL of PBS and simultaneously stained with 200 μL of propidium iodide (50 μL/mL; Sigma-Aldrich) and incubated with 20 μL of RNase (1 mg/mL; Sigma-Aldrich) in a 37°C water bath for 15–20 min. The percentage of cells in each phase of the cell cycle was determined using a FACStation (FV500, Beckman Coulter, Brea, CA, USA) and analyzed using the Kaluza^®^ Flow Analysis Software. Each experiment was repeated in triplicate.

For apoptosis analyses, 1 × 10^5^ cells were stained with Annexin V and propidium iodide using an Annexin V-APC kit (BD Pharmingen^™^ catalog number 550474, BD Biosciences, Franklin Lakes, NJ, USA) and analyzed with a FACStation equipped with CellQuest software. Each experiment was repeated in triplicate.

### β-galactosidase senescence assays

A total of 1 × 10^6^ A2780 cells, 4 × 10^5^ OVCA433 cells, and 4 × 10^5^ OVCA429 cells were cultured in a 6-cm dish and incubated for 3 days in RPMI-1640 medium supplemented with 10% FBS. When the cells reached approximately 80% confluence, they were fixed and incubated with a freshly prepared senescence-associated β-galactosidase staining solution in the dark at 37°C overnight. The percentage of cells that were positive for β-galactosidase activity was determined by counting the number of blue cells in 10 fields at 20 × magnification. Each experiment was repeated in triplicate.

### Western blotting

Western blotting was performed as previously described [34]. Primary antibodies against the following proteins were obtained from Santa Cruz Biotechnology (Santa Cruz, CA, USA): P14^ARF^, P15^INK4B^, P16^INK4A^, Bcl-2, survivin, caspase-9, caspase-3, and caspase-7. β-actin (A2228, Sigma-Aldrich) was used as a loading control. The secondary antibodies were F(ab)2 fragments of donkey anti-mouse immunoglobulin or donkey anti-rabbit immunoglobulin linked to horseradish peroxidase (Cell Signaling Technology, Beverly, MA, USA). Immunoblotting reagents from an electrochemiluminescence kit were used (Amersham Biosciences, Uppsala, Sweden).

### Xenograft tumors in nude mice

The animal experiments were approved by the Institutional Animal Care and Use Committee of Fudan University and were performed according to the institutional guidelines and protocols. Female BALB/c athymic nude mice (4–6 weeks old weighing 20–22 g) were provided by the Department of Laboratory Animals of Fudan University and housed in a pathogen-free animal facility. To generate tumor growth *in vivo*, 1 × 10^6^ A2780-KD1, A2780-KD2, and A2780-NC cells were subcutaneously injected into the mice (*n* = 6 for each cell line). The tumor volume was calculated as previously described [45]. Once a tumor reached 1.0 cm in diameter, the mice were euthanized and the tumors weighed.

### Statistical analyses

All statistical analyses were performed using SPSS for Windows v.16.0 (SPSS, Chicago, IL, USA). Continuous data were analyzed using an independent *t*-test between two groups. Categorical data were analyzed using the chi-square test or Fisher's exact test as appropriate. OS rates were calculated using the Kaplan-Meier method and the log-rank test for comparisons. Multivariate survival analyses were performed on all factors that were significant in univariate analyses using the Cox regression model. *P* values < 0.05 were considered significant (*P* < 0.05).

## SUPPLEMENTARY FIGURES


