# Supplementary material for: The long non-coding RNA ANRIL promotes proliferation and cell cycle progression and inhibits apoptosis and senescence in epithelial ovarian cancer
Source: Oncotarget. 2016 Apr 15;7(22):32478–92. doi: 10.18632/oncotarget.8744 (PMC5078027; doi:10.18632/oncotarget.8744)
Supplement: Supplementary file 1 [file oncotarget-07-32478-s001.pdf]

The long non-coding RNA ANRIL promotes proliferation and cell cycle progression and inhibits apoptosis and senescence in epithelial ovarian cancer

Supplementary Materials

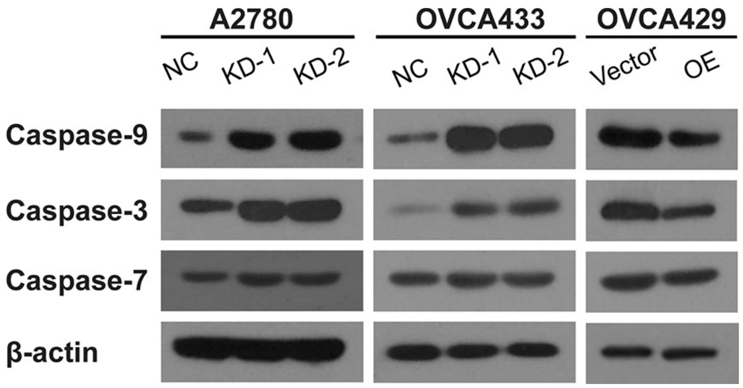

Supplementary Figure S1: Knockdown and overexpression of ANRIL alters caspase-9 and caspase-3 expression. Western blots showing that ANRIL knockdown increases caspase-9 and caspase-3 levels in A2780 and OVCA433 cells, while ANRIL overexpression decreases caspase-9 and caspase-3 levels in OVCA429 cells.
